# Impact of SARS-CoV-2 Infection and Vaccination on Pregnancy Outcome and Passive Neonatal Immunity

**DOI:** 10.3390/cells14221812

**Published:** 2025-11-19

**Authors:** Gina Marie Uehre, Valeriia Grabar, Evelin Grage-Griebenow, Oliver Klemens, Laura Scholz, Nils Hoymann, Suzan Alboradi, Atanas Ignatov, Svetlana Tchaikovski, Mandy Busse

**Affiliations:** 1 Experimental Obstetrics and Gynecology, Medical Faculty, Otto-von-Guericke University, 39108 Magdeburg, Germany; gina.uehre@med.ovgu.de (G.M.U.); laura2.scholz@st.ovgu.de (L.S.); suzan.alboradi@hotmail.com (S.A.); 2 University Hospital for Obstetrics and Gynecology, Medical Faculty, Otto-von-Guericke University, 39108 Magdeburg, Germanyatanas.ignatov@med.ovgu.de (A.I.); svetlana.tchaikovski@uk-brandenburg.de (S.T.); 3 Institute for Experimental Immunology, Affiliated with EUROIMMUN AG Medizinische Labordiagnostika AG, 23560 Luebeck, Germany; e.grage-griebenow@euroimmun.de (E.G.-G.); o.klemens@euroimmun.de (O.K.); 4 University Clinic for Obstetrics and Gynecology, Medical Faculty, Brandenburg Medical School Theodor Fontane, 14770 Brandenburg an der Havel, Germany

**Keywords:** SARS-CoV-2, infection, mRNA vaccination, pregnancy, umbilical cord blood

## Abstract

**Background**: During the SARS-CoV-2 pandemic, many women were infected or received vaccinations against the virus before or during their pregnancy. Little is known about the possible consequences of vaccination or infection on obstetric outcomes, as well as antibody levels against other infectious agents, such as the TORCH pathogens. **Methods**: A total of 136 pregnant women were included in our study between March 2022 and February 2024. The concentrations of antibodies against nucleocapsid (NCP), the spike protein of SARS-CoV-2, as well as IgG and IgM antibodies against TORCH, were assessed in the maternal and umbilical cord blood. **Results**: The patients were grouped into the following categories according to responses given in the questionnaire and antibody titer: controls (neither infected nor vaccinated; N = 17), infected only (N = 35), vaccinated only (N = 21), acutely infected (N = 15), and both vaccinated and experienced a COVID-19 infection (N = 47). No differences between the groups in terms of pregnancy outcomes were found. The presence of IgG antibodies against NCP or spike protein in maternal blood was dependent on the patient’s vaccination status or previous infection, correlating with that in cord blood. The level of maternal IgG against spike protein correlated negatively with TORCH antibodies. **Conclusions**: The present study demonstrates the infection- and vaccination-dependent formation of SARS-CoV-2-specific antibodies in the mother and their transfer to the unborn child. Further studies are necessary to investigate the interaction between SARS-CoV-2-specific antibodies and antibodies formed by infection (e.g., CMV) or vaccination against other pathogens in the mother and transmitted transplacentally to the unborn child.

## 1. Introduction

The World Health Organization (WHO) declared the outbreak of the novel coronavirus disease (COVID-19) a global pandemic on 11 March 2020 [1]. The virus responsible for COVID-19 was termed severe acute respiratory syndrome coronavirus 2 (SARS-CoV-2). This positive single-stranded RNA virus consists of four major structural proteins, called spike (S), membrane (M), envelope (E) and nucleocapsid (NCP), as well as sixteen other non-structural proteins [2]. SARS-CoV-2 initiates infection through binding the spike protein to the host angiotensin-converting enzyme 2 (ACE2) receptor. Given its role in viral entry, the spike protein is an important target for neutralizing antibodies and has been widely used in the design of vaccines against SARS-CoV-2, e.g., mRNA-based vaccines developed by Pfizer/BioNTech and Moderna [3].

Early in the pandemic, it became evident that pregnant women represented a group particularly vulnerable to SARS-CoV-2 infection, with an increased risk of pre-eclampsia, stillbirth, and maternal and neonatal mortality [4,5]. The risk of preterm birth and neonatal intensive care unit admission due to prematurity was increased in patients with moderate-to-severe infections across all SARS-CoV-2 variant periods. Furthermore, infants under six months of age remain at elevated risk of acute COVID-19-related complications [6]. To reduce maternal and neonatal morbidity and mortality, early public health recommendations in Germany advised the administration of at least three doses of an mRNA-based vaccine before or during pregnancy. Vaccination during pregnancy has been demonstrated to reduce incidence of maternal and fetal complications following SARS-CoV-2 infection [7,8].

The maternal–fetal interface functions as a critical barrier, restricting vertical transmission of pathogens and protecting the fetus from maternal infections. Interestingly, even though the placenta expresses ACE2, the primary cellular receptor for SARS-CoV-2, confirmed placental infection appears to be rare. The vertical transmission of SARS-CoV-2 is characterized by the presence of specific anti-SARS-CoV-2 immunoglobulins M (IgM) and G (IgG) antibodies in amniotic fluid, along with an extensive fetal inflammatory response [9]. During SARS-CoV-2 infection, IgA and IgM are the first antibodies to be produced in response to the virus, typically becoming detectable within five to seven days after symptom onset. Both serve as an early marker of acute infection, but IgM does not cross the placental barrier due to its large molecular size. Therefore, the detection of anti-SARS-CoV-2 IgM in the fetal compartment is indicative of a fetal immune response.

In contrast, maternal IgG—either acquired through natural infection or vaccination—crosses the placenta and provides the primary form of passive immunity to the fetus. Maternal IgG can be detected in cord blood [10,11,12], which is associated with a longer COVID-19-free period in neonates [13] and contributes to reducing neonatal hospitalization in the first six months of life [14].

SARS-CoV-2 has also been considered a potential “TORCH” infection [9]. The term “TORCH” refers to a group of congenital infections, including Toxoplasmosis, Others (such as syphilis, hepatitis B), Rubella virus, Cytomegalovirus, Herpes simplex viruses 1 and 2, which are known to cause harm to the developing fetus. The severity of the fetal damage largely depends on the timing of maternal infection during pregnancy. Infections occurring in the first trimester are typically associated with the most serious adverse effects, including embryopathy, fetopathy, abortion, preterm birth, or intrauterine growth restriction. The transplacental transfer of maternal IgG specific to the TORCH pathogens provides the fetus or newborn with protective immunity against these infections.

Currently, there is a paucity of studies investigating a potential impact of maternal anti-SARS-CoV-2 antibody levels induced by either vaccination or infection on the transplacental transfer of TORCH-specific antibodies [15,16].

In this study, we aimed to quantify the levels of anti-spike and anti-NCP IgG antibodies in maternal and cord blood, and to examine their correlation with TORCH antibody presence. In addition, we analyzed pregnancy outcomes (e.g., abnormal fetal ultrasound, blood loss, or obstetric complications during delivery) and neonatal health in relation to SARS-CoV-2 infection and/or vaccination during pregnancy.

## 2. Materials and Methods

### 2.1. Sample Collection

This study was approved by the Ethics Committee of the Otto-von-Guericke University Medical Faculty (approval number: 19/22). All patients provided written informed consent before participation. Information regarding previous SARS-CoV-2 infection or vaccination before and during pregnancy was collected via a questionnaire. Following their admission to the hospital, patients were screened for current SARS-CoV-2 infection via antigen rapid testing or PCR.

Maternal blood samples were collected shortly before delivery at the University Hospital for Obstetrics and Gynecology Magdeburg, Germany, from March 2022 until February 2024. Umbilical cord blood samples were obtained immediately after delivery. Serum and plasma were prepared immediately after sample collection and stored at −80 °C until analysis.

### 2.2. Anti-SARS-CoV-2 NCP ELISA (IgG)

The Anti-NCP ELISA (IgG) (Euroimmun, Lübeck, Germany) was performed according to the manufacturer’s instructions for the semiquantitative assessment of antibodies against the nucleocapsid. Briefly, samples were diluted 1:101 with sample buffer and incubated for 1h at 37 °C ± 1 °C in the coated reagent wells. Samples were washed 3 times prior to 30 min incubation with peroxidase-labeled anti-human IgG, washed 3 times and incubated for 15 min with chromogen/substrate solution in the dark. The reaction was stopped after 15 min with 0.5 M sulphuric acid. Photometric measurement was performed at a wavelength of 450 nm. Anti-NCP ratio was calculated through division of sample extinction and calibrator extinction. A ratio above 1.1 was considered as positive; a ratio ≥0.8 to <1.1 was considered a borderline value that cannot be considered positive, in accordance with the manufacturer’s instruction, resulting in the exclusion of 16 samples of maternal blood and 34 umbilical cord blood that could not be considered clearly positive or negative.

### 2.3. Anti-SARS-CoV-2 QuantiVac ELISA (IgG)

The Anti-SARS-CoV-2 QuantiVac ELISA (IgG) (EUROIMMUN Medizinische Labordiagnostika AG, Lübeck, Germany; hereafter referred to “Euroimmun”) was performed according to the manufacturer’s instructions. Briefly, samples were diluted 1:101/1:1.001 or 1:10.001 with sample buffer and incubated for 1 h at 37 °C ± 1 °C in the coated reagent wells. Samples were washed 3 times prior to 30 min incubation at 37 °C ± 1 °C with peroxidase-labeled anti-human IgG, again washed 3 times and incubated for 15 min with chromogen/substrate solution in the dark. The reaction was stopped after 30 min with 0.5 M sulphuric acid. Photometric measurement was performed at a wavelength of 450 nm. The concentration of the patient’s antibodies was calculated with a standard curve generated with the six calibrators provided by the manufacturer.

### 2.4. EUROLINE Anti-TO.R.C.H.Profile (IgG) and (IgM)

Maternal and umbilical cord blood serum samples were analyzed for IgM and IgG antibodies against *Toxoplasma gondii* tachyzoites, Rubella virus strain HPV-77, Cytomegalovirus phosphoproteins, glycoprotein C1 of Herpes simplex virus type 1 and glycoprotein G2 of Herpes simplex virus type 2 with the use of the EUROLINE Anti-TO.R.C.H. Profile kits (IgM, IgG, Euroimmun, Lübeck, Germany) according to the manufacturer’s recommendations.

The EUROLineScan software 3.4.37 (Euroimmun, Lübeck, Germany) was used to evaluate the incubated test strips. A correct detection of IgG/IgM class antibodies against TORCH antigens is only indicated in case of both positive reaction of the control band and positive reaction of the IgG/IgM band.

### 2.5. Statistics

Statistical analysis was performed with GraphPad Prism 8 (Dotmatics, Boston, MA, USA). The patient’s characteristics were assessed regarding normality via Shapiro–Wilk Test. Differences between the groups were analyzed using One-Way-ANOVA for normally distributed variables or the Kruskal–Wallis Test for non-parametric data, followed by Dunnet’s or Dunn’s multiple comparison tests. Correlations were evaluated by Spearman correlation. Significance was defined as follows: * *p* < 0.05; ** *p* < 0.01; *** *p* < 0.001; **** *p* < 0.0001.

## 3. Results

### 3.1. Patient Cohort

A total of 136 pregnant women were recruited for this study immediately before their admission to our hospital for labor and delivery. The inclusion criteria encompassed informed consent, completion of 37 weeks of gestation, and maternal age ≥18 years. Exclusion criteria included pregnancy complications such as pre-eclampsia, HELLP syndrome, or premature birth. Participants completed a standardized questionnaire on their SARS-CoV-2 vaccination status (i.e., number, type, and dates of doses received) and any prior SARS-CoV-2 infection (including diagnostic method, such as rapid antigen testing or PCR, and corresponding dates). The distributed virus variants that were observed during the course of the study in Germany comprised omicron variants BA.4/5 [17,18].

Based on the information collected, the cohort was stratified into five groups:

(1) The control group comprised 17 women with no history of SARS-CoV-2 vaccination or confirmed infection.
(2) The vaccinated group included 35 women who had received at least one dose of SARS-CoV-2 vaccine during pregnancy but had no documented history of infection.
(3) Twenty-one patients with confirmed SARS-CoV-2 infection during pregnancy but without vaccination were considered the infected group.
(4) Fifteen women who tested positive for SARS-CoV-2 by PCR at the time of the delivery were categorized as the acutely infected group (ten of them had been vaccinated).
(5) The vaccinated and infected group included 47 women who had received at least one dose of vaccine and had a previous confirmed SARS-CoV-2 infection during pregnancy.

No statistically significant differences were identified in the maternal and neonatal parameters collected in relation to the infection or vaccination status of the women (Table 1). A further sub-division of the acutely infected group was conducted according to the subjects’ vaccination status. However, no differences were found between these subgroups in any of the parameters.

Among women with a documented history of SARS-CoV-2 infection, 42.6% (29 of 68) were infected during the third trimester of pregnancy (Table S1). Overall, 66.2% of the study participants were vaccinated against SARS-CoV-2. The majority of them received their vaccinations during the second trimester of pregnancy (Table S2). Furthermore, the levels of anti-spike IgG and the presence of anti-NCP IgG in maternal and umbilical cord blood were correlated with the most recent time point of maternal vaccination or infection. The most recent time point of maternal vaccination during pregnancy (trimester) exhibited a significant correlation with maternal and umbilical cord blood anti-spike IgG level. Similarly, the most recent time point of maternal infection during pregnancy (trimester) demonstrated a significant correlation with the presence of anti-NCP antibodies in maternal and umbilical cord blood (Table S3).

### 3.2. Anti-Spike Antibodies in Maternal and Umbilical Cord Blood

**Figure 1:**
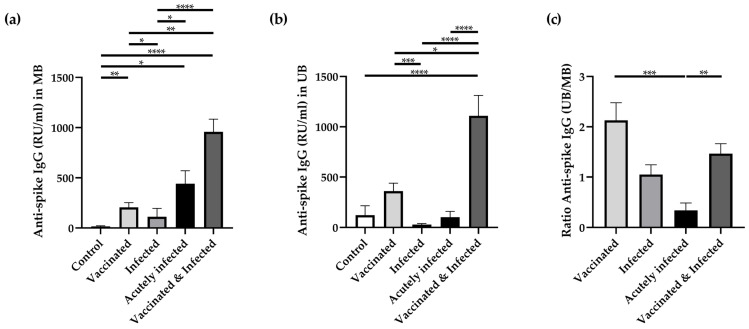
**Levels of anti-spike IgG (in RU/mL) amongst the study groups with respect to SARS-CoV-2 infection or vaccination during pregnancy.** The presence of antibodies was evaluated in two distinct biological specimens—maternal blood (MB; **a**) and umbilical cord blood (UB; **b**)—using the Euroimmun QuantiVac ELISA (Euroimmun, Lübeck, Germany). The transfer ratio (**c**) was calculated as the anti-spike IgG level (in RU/mL) in UB divided by that in MB for all available sample pairs. The data were analyzed using the Kruskal–Wallis test and are shown as mean ± SEM. * *p* < 0.05; ** *p* < 0.01; *** *p* < 0.001; **** *p* < 0.0001.

The development of antibodies against the spike protein of SARS-CoV-2 can occur either after vaccination with a spike protein-based immunogen or following a natural infection with the virus. In the maternal and umbilical cord blood samples from the control group, the presence of anti-spike antibodies was negligible (Figure 1a,b). Anti-spike IgG levels in both maternal and umbilical cord blood were elevated in women with a history of SARS-CoV-2 infection or vaccination. The highest antibody levels were observed in patients who had been both vaccinated and previously infected. Notably, women who experienced SARS-CoV-2 infection during pregnancy exhibited lower anti-spike antibody concentrations compared to both vaccinated individuals and those with acute infection. Interestingly, in the acutely infected group, anti-spike antibody levels were consistently higher in maternal blood compared to those in the corresponding umbilical cord blood (Figure 1a,b).

Additionally, we calculated the ratios between the anti-spike antibody levels in umbilical cord and those in the corresponding maternal blood (Figure 1c). Values greater than 1 indicate higher antibody levels in fetal blood as compared to the mother (e.g., 2.1 in the vaccinated group). In contrast, ratios less than 1 reflect higher maternal antibody concentrations as compared to the corresponding umbilical cord blood (0.3 in the acutely infected group).

### 3.3. Anti-NCP Antibodies in Maternal and Cord Blood

In the groups with previous or current (acute) SARS-CoV-2 infection with or without vaccination, antibodies against the SARS-CoV-2 NCP could be detected in between 36.4% and 60% of maternal and umbilical cord blood samples, respectively (Table 2). Notably, anti-NCP IgG were also detected in 15 maternal and umbilical cord blood samples without reported previous SARS-CoV-2 infection (control and vaccinated group). Positive correlations were identified between the maternal and umbilical cord blood anti-NCP IgG ratios in all groups with a history of SARS-CoV-2 infection or vaccination (Table 2). While there was an absence of statistically significant transfer ratios in the control group, notable correlations were identified in the following cohorts: the vaccinated-only (*p* = 0.0001), infected-only (*p* = 0.0335), acutely infected (*p* = 0.0030), and vaccinated and infected patients (*p* = 0.0005). While there was an absence of statistically significant transfer ratios in the control group, notable correlations were identified in the following cohorts: the vaccinated-only (*p* = 0.0001), infected-only (*p* = 0.0335), acutely infected (*p* = 0.0030), and vaccinated and infected patients (*p* = 0.0005). The mean transfer ratio of anti-NCP antibodies from MB to UB was 1.413 in the infection-only group, 1.128 in the vaccinated/infected group and 0.5772 in the acutely infected group (*p* = 0.0079 vs. infected-only patients and *p* = 0.0118 vs. vaccinated and infected group; Figure S1).

### 3.4. Correlation Between Anti-NCP and Anti-Spike Protein IgG

The concentrations of maternal anti-NCP IgG (*p* = 0.0010) and anti-spike IgG (*p* = 0.0279) were correlated with confirmed SARS-CoV-2 infection. Additionally, a positive correlation was observed between the number of maternal vaccine doses and the anti-spike IgG levels in maternal and umbilical cord blood (both *p* < 0.0001; Table 3). In contrast, the anti-NCP-specific IgG levels were unaffected by vaccination status (Table 3).

### 3.5. Correlation Between Anti-NCP Antibody Presence and Anti-Spike Antibodies with the Presence of TORCH Antibodies in Maternal and Umbilical Cord Blood

Finally, the potential impact of SARS-CoV-2 infection and vaccination on the presence of TORCH antibodies in maternal and cord blood was investigated. IgM antibodies against *Toxoplasma gondii* and CMV were detected in 14.2% and 3% of all tested maternal blood samples, respectively, but not in the umbilical cord blood samples (Table S4). Detection rates of IgG against TORCH pathogens in maternal and umbilical cord blood varied by pathogen, ranging from 0% for HSV-2 to 100% for Rubella virus. The distribution of positive TORCH serology was rather similar across the study groups, except for anti-CMV IgG, which was significantly more frequently present in maternal blood of the controls and infected compared to the vaccinated, acutely infected, and vaccinated and infected group (Figure S2).

In contrast, statistically significant correlations were identified between maternal and umbilical cord IgG levels for each detectable TORCH antigen (all *p* < 0.0001, Table S5). While we could not detect any correlation between the presence of anti-spike antibodies in maternal blood and antibodies against any of TORCH antigens in either maternal or umbilical cord blood, there was a negative correlation between the presence of anti-CMV IgG in maternal and umbilical cord blood with the levels of anti-spike antibodies in maternal blood. In contrast, the positive maternal serology for anti-NCP IgG correlated with the presence of anti- *Toxoplasma* and anti-HSV-2 IgG in umbilical cord blood (Table 4).

## 4. Discussion

Following the SARS-CoV-2 pandemic, numerous questions have remained unanswered, particularly concerning pregnant women who were infected with the virus or received vaccination during pregnancy. The recommendation to vaccinate during pregnancy was based on a substantial body of evidence that demonstrated an association between SARS-CoV-2 infection during pregnancy and an increased risk of adverse pregnancy outcomes, including preeclampsia, stillbirth, and preterm birth [19,20,21,22,23].

The objective of this study was to evaluate the impact of SARS-CoV-2 infection and immunization on pregnant women delivering at term, thereby minimizing confounding potential factors such as those related to preterm birth or preeclampsia. It is important to note that, in our study, all women who tested positive for SARS-CoV-2 at the time of delivery exhibited only mild symptoms of the disease. In contrast to several other reports, our results indicated that neither infection nor vaccination had a significant effect on pregnancy outcomes. Some published studies have shown that pregnant women infected with SARS-CoV-2 during pregnancy exhibit elevated uterine artery Doppler indices, suggesting potential placental dysfunction and an increased risk of adverse pregnancy outcomes [24,25]. We were unable to replicate these findings in any of the study groups. Similarly, we found no evidence of increased incidence of intrauterine growth restriction following SARS-CoV-2 infection during pregnancy, contrary to previous reports [26]. These discrepancies could potentially be explained by the limited sample size, or infections involving less pathogenic SARS-CoV-2 variants. Additionally, if SARS-CoV-induced intrauterine growth restriction is mediated through preeclampsia-like mechanisms or by triggering preeclampsia itself, this discrepancy could also reflect a selection bias, since patients with preeclampsia were excluded from our study. As reported in large cohort studies as well as meta-analysis studies, the incidence of intrauterine growth restriction (IUGR) is estimated at 2–3% in asymptomatic cases or cases presenting with mild symptoms. This rate is similar to or only slightly higher than the rate observed in the general population [27,28,29,30]. The presence of symptoms or a long-term course of SARS-CoV-2 infection, particularly in cases of severe disease, has been demonstrated to heighten the likelihood of IUGR and associated complications [31].

The incidence of acute SARS-CoV-2 infection among pregnant women has varied across Europe, with rates reported at 0.3% in summer 2020, rising to over 3% by the end of 2021 [32,33], and reaching 9.5% in the U.S. through 2022 [34]. In Germany, there is a paucity of data regarding the incidence of SARS-CoV-2 infection among pregnant women, particularly at the time of delivery.

An important objective of this study was to assess the presence and persistence of specific anti-SARS-CoV-2 immunoglobulins following vaccination and infection in maternal and cord blood during pregnancy. The vaccine’s efficacy in non-pregnant individuals has been reported to persist for six to twelve months, with stable receptor-binding domain Ig levels [35,36,37], and a stronger, more sustained IgG response following booster vaccinations, detectable for up to 50 weeks [38]. Consistent with this, our study demonstrated a positive correlation between the number of vaccine doses and anti-spike IgG levels in both maternal and cord blood, reinforcing the rationale for booster vaccinations [39]. Furthermore, we confirmed a previous report [40] on the highest anti-spike IgG levels in maternal and cord blood from women who had both been vaccinated and infected as compared to those who had either been vaccinated or infected. Therefore, the present study provides further evidence for the safety and immunological benefits of SARS-CoV-2 vaccination during pregnancy, as well as for the benefits of receiving additional vaccinations against the virus, which have been shown to induce long-term antibody persistence [41,42,43].

Interestingly, the cord-to-maternal anti-spike IgG ratio was highest in women who had only been vaccinated. In contrast, acutely infected mothers exhibited lower fetal anti-spike IgG levels relative to maternal levels. Reduced placental transfer of SARS-CoV-2-specific antibodies has previously been reported, particularly compared to other infections, such as influenza or pertussis [44]. This phenomenon appears to be limited to de novo-produced antibodies following infection during the third trimester of pregnancy. Other studies have identified placental transfer ratios ranging from 0.5 to 1.6 following vaccination against SARS-CoV-2, with a strong correlation between maternal and cord anti-spike IgG levels [38,45,46]. Studies on maternal-neonate dyads have further demonstrated that spike-specific antibodies detected in cord blood exhibit neutralizing activity against SARS-CoV-2 variants comparable to that observed in maternal antibodies.

Previous studies have demonstrated that anti-NCP antibodies are detectable in all patients with SARS-CoV-2 infection, with levels typically declining approximately 20 days post-infection but remaining detectable for up to 240 days [39,47]. However, the absence of a SARS-CoV-2-specific IgG response has also been reported in 5% of cases for anti-spike IgG and in up to 20% for both anti-NCP and anti-spike IgG in acutely infected pregnant women [48]. In the present study, fewer than 40% of women with confirmed SARS-CoV-2 infection had detectable anti-NCP IgG (31/81). This relatively low seropositivity may be attributed to the pregnancy-related immune modulation, particularly the shift towards immune tolerance, which can alter the adaptive immune system.

Interestingly, anti-NCP IgG was detected in a few cord blood samples even in the absence of measurable levels in the corresponding maternal samples. Since isolated fetal infection is considered highly unlikely, this finding suggests that maternal anti-NCP antibodies were transferred across the placenta before declining below the detection threshold in maternal serum. Additionally, 12% of women who did not report prior infection, i.e., the control and vaccinated group, had detectable anti-NCP IgG in maternal serum and umbilical cord blood serum. This suggests a previous undiagnosed or asymptomatic SARS-CoV-2 infection, which likely resulted in the development of specific antibodies. Similar observations regarding unrecognized infections in pregnant populations have been reported elsewhere [49]. Another explanation is rare crossreactivities with NCP IgG directed against other (older) Coronaviruses [50]. As expected, the number of vaccination doses had no impact on the detectability of anti-NCP antibody, since NCP is not an antigenic target of current SARS-CoV-2 vaccines.

In addition, our study demonstrated that the presence of maternal IgG against TORCH pathogens in maternal blood reliably predicted their presence in cord blood, consistent with previous findings [51,52]. Independent of SARS-CoV-2 infection, pandemic-related restrictions had an indirect impact on the incidence of congenital infections. For instance, a substantial decline in the rate of congenital CMV infections was reported in pregnant women during 2020 and 2021 [53,54]. Additionally, SARS-CoV-2-specific IgG might also influence the transplacental transport of anti-TORCH antibodies. However, the presence of anti-NCP IgG in maternal blood did affect the rate of detected IgG against TORCH pathogens, i.e., *Toxoplasma gondii* and HSV-2 in umbilical cord blood. A previously published study found no alterations in the transplacental transfer of HSV-specific IgG in mothers who tested positive for SARS-CoV-2 antibodies [46]. In our study cohort, maternal serological levels of SARS-CoV-2 anti-spike IgG had a significant impact on the detection rates of antibodies against the TORCH pathogens CMV in maternal and umbilical cord blood. In line with this, a significantly reduced presence of anti-CMV antibodies was detected in the vaccinated and infected group. However, maternal anti-spike IgG positivity was associated with reduced detection rates of CMV, HSV-1, and HSV-2 IgG in both maternal and cord blood. Conversely, the reduced anti-HSV-1 and HSV-2 antibody presence suggests that the maternal–fetal transfer process might have undergone a reduction due to elevated anti-spike concentrations. Research on IgG subclass transfer during pregnancy indicates that IgG1 is transferred most efficiently to the fetus, followed by IgG3, IgG4, and IgG2 [45]. Furthermore, the study demonstrated that vaccination against influenza was associated with increased IgG4 levels in both mothers and neonates. However, it did not affect transfer ratios. As Wilcox et al. [55] described, elevated concentrations of vaccine-specific antibodies could possibly lead to a diminished proportion of maternal IgG being transferred across the placenta to the infant, resulting in a reduced transplacental transfer ratio due to competition among FcRn receptors. However, these findings have yet to be substantiated in the context of TORCH antibodies. Collectively, direct studies on the transfer of TORCH-specific IgG subclasses, as well as the influence of infections or vaccinations on this process, remain limited and necessitate further investigation.

Taken together, our findings confirm the efficient maternal–fetal transfer of SARS-CoV-2-specific anti-spike and anti-NCP IgG following infection or vaccination during pregnancy. The magnitude of the antibody response was positively associated with the number of vaccine doses received. The potential influence of SARS-CoV-2-induced immunity on the transplacental transfer of TORCH-specific antibodies appears to be marginal and remains to be further elucidated.

## Figures and Tables

**Table 1:** **Demographic characteristics and obstetric outcomes.** All events deviating from the normal course of birth, including perineal tears, postpartal hemorrhage, umbilical cord entanglements and insufficient contractions, were considered to be obstetric complications. Neonatal complications observed included icterus in the newborn, and difficulties in adaptation that necessitated intensive care for the newborn. All normally distributed parameters are expressed as average ± standard deviation.

	Control (n = 17)	Vaccinated (n = 35)	Infected (n = 21)	Acutely Infected (n = 15)	Vaccinated and Infected (n = 47)	*p* Value
**Maternal age, years**	30.5 ± 7.4	31.2 ± 5.4	29.3 ± 6.1	31.2 ± 4.8	32.1 ± 6.2	0.4975
**Gestational age, weeks**	38.5 ± 1.5	39.4 ± 1.6	38.3 ± 1.5	39.3 ± 1.2	38.9 ± 1.7	0.0503
**Twin pregnancies % (N)**	11.8% (2)	0	19.0% (4)	0	2.1% (1)	
**Gravidity**	2.9 ± 2.0	2.1 ± 1.5	3.0 ± 1.9	2.3 ± 1.5	2.4 ± 1.6	0.2397
**Parity**	2.5 ± 1.9	1.7 ± 1	2.2 ± 1.3	1.9 ± 1.2	1.8 ± 1	0.3866
**Abnormal fetal ultrasound, % (N)**	11.8% (2)	8.6% (3)	19.0% (4)	20.0% (3)	17.0% (8)	0.6672
**Cesarean section, % (N)**	41.2% (7)	37.1% (13)	42.9% (9)	46.7% (7)	55.3% (26)	0.6208
**Blood loss, mL**	364 ± 122	334 ± 119	376 ± 175	457 ± 312	393 ± 138	0.3935
**Obstetric complications, % (N)**	23.5% (4)	62.9% (22)	42.9% (9)	60.0% (9)	40.0% (19)	0.0546
**Neonatal complications, % (N)**	29.4% (5)	42.9% (15)	28.6% (6)	40.0% (4)	36.2% (17)	0.5611
**Birth weight, g**	3321 ± 609	3465 ± 447	3202 ± 533	3576 ± 625	3544 ± 523	0.0684
**Neonatal weight at discharge, g**	3075 ± 517	3279 ± 403	2942 ± 495	3375 ± 567	3337 ± 444	0.0078
**Birth length, cm**	50.3 ± 3	51.4 ± 2.2	50.4 ± 2.7	52.7 ± 2.2	51.2 ± 3.8	0.0483
UB * pH	7.31 ± 0.09	7.27 ± 0.09	7.30 ± 0.06	7.25 ± 0.07	7.26 ± 0.09	0.0793
UB * Base excess	−1.2 ± 4.1	−1.4 ± 4.5	−1.2 ± 2.5	−2.6 ± 3.3	−2.2 ± 3.6	0.5527
**APGAR 1 min**	9.2	9	8.2	9.2	8.6	0.9707
**APGAR 5 min**	9.7	9.3	9.6	9.9	9.4	0.9029
**APGAR 10 min**	9.9	9.7	9.9	10	9.7	0.3808

* UB: umbilical cord blood.

**Table 2:** Disparities in anti-NCP IgG positivity amongst the study groups with respect to vaccination or infection during pregnancy. The data presented herein are expressed as the number (N) of positive blood samples per total number of samples investigated, alongside the respective percentages. The correlation was determined using the Spearman method, with the resulting r and *p* values presented. MB, maternal blood; UB: umbilical cord blood; NCP, nucleocapsid.

	Control	Vaccinated	Infected	Acutely Infected	Vaccinated and Infected
**Positive anti-NCP IgG in MB (%; N)**	12.5% (2/15)	12.5% (4/32)	44.4% (8/18)	50% (7/14)	40% (16/40)
**Positive anti-NCP IgG in UB (%; N)**	33.3% (3/9)	22.2% (6/27)	47.4% (9/19)	36.4% (4/11)	60% (21/35)
**Correlation MB/UB Spearman r**	0.7698	0.6633	0.4893	1.000	0.5372
**Correlation MB/UB** ***p* value**	0.0545	0.0001	0.0335	0.0030	0.0005

**Table 3:** Correlation between anti-spike and NCP IgG levels in maternal and umbilical cord blood with confirmed SARS-CoV-2 infection during pregnancy and the number of vaccinations. The correlations were determined between anti-spike IgG in RU/mL or anti-NCP IgG as ratio and a positive PCR-based SARS-CoV-2 test during pregnancy or number of vaccinations using the Spearman method, and the correlation coefficients (r) with corresponding *p* values presented. MB, maternal blood; UB, umbilical cord blood; NCP, nucleocapsid.

	Confirmed SARS-CoV-2 Infection		Number of Vaccine Doses	
	r	*p*	r	*p*
**MB anti-NCP IgG**	0.3048	0.0010	−0.1472	0.0986
**MB anti-spike IgG**	0.2107	0.0279	0.6273	<0.0001
**UB anti-NCP IgG**	0.1538	0.1389	−0.1327	0.1772
**UB anti-spike IgG**	−0.05537	0.6105	0.6504	<0.0001

**Table 4:** Correlation between the appearance of IgG TORCH antibodies in maternal and umbilical cord blood with SARS-CoV-2 spike and NCP antibodies in maternal serum. Shown are the Spearman r and *p* values. MB, maternal blood; UB, umbilical cord blood; NCP, nucleocapsid.

TORCH	MB Anti-NCP IgG+		MB Anti-Spike IgG+		MB Anti-Spike IgG Level	
	r	*p*	r	*p*	r	*p*
**MB *Toxoplasma gondii* IgG**	0.1515	0.0879	0.04043	0.6597	0.05624	0.5401
**MB Rubella virus IgG**	0.01501	0.8696	0.06571	0.4834	0.09489	0.3110
**MB Cytomegalovirus IgG**	−0.1062	0.2424	−0.1242	0.1822	−0.2647	0.0039
**MB Herpes simplex virus I IgG**	−0.01627	0.8554	−0.004460	0.9613	−0.1602	0.0792
**MB Herpes simplex virus II IgG**	0.2043	0.0218	−0.08373	0.3653	−0.09920	0.2831
**UB *Toxoplasma gondii* IgG**	0.2316	0.0218	0.1104	0.2920	0.05221	0.6192
**UB Rubella virus IgG**	-	-	-	-	-	-
**UB Cytomegalovirus IgG**	−0.1277	0.2200	−0.1592	0.1361	−0.2883	0.0062
**UB Herpes simplex virus I IgG**	0.02185	0.8318	−0.01850	0.0582	−0.09920	0.2831
**UB Herpes simplex virus II IgG**	0.3379	0.0007	−0.1055	0.3169	−0.07874	0.4556

## Data Availability

The data are unavailable due to ethical restrictions.

## Supplementary Materials

The following supporting information can be downloaded at: https://www.mdpi.com/article/10.3390/cells14221812/s1, Figure S1. NCP IgG transfer ratio between maternal blood to umbilical cord blood in previously or currently infected, as well as vaccinated and infected, groups. Data were analyzed using the Kruskal–Wallis test and shown as mean ± SEM. * *p* < 0.05 ** *p* < 0.01. Figure S2. Positivity for anti-CMV IgG in maternal blood samples across the study groups. Data were analyzed using the Kruskal–Wallis test and shown as mean ± SEM. * *p* < 0.05. Table S1. Number of pregnant women by the time of infection; Table S2. Number of pregnant women by the time of vaccination; Table S3. Levels of anti-spike and presence of anti-NCP antibodies in maternal and umbilical cord blood in correlation with the latest time point of maternal vaccination or infection; Table S4. Presence of anti-TORCH antibodies in maternal and umbilical cord blood. The number of patients positive for anti-CMV antibodies from the total number of patients and the corresponding percentages are given. MB, maternal blood; UB, umbilical cord blood. Table S5. Correlation of appearance of anti-TORCH IgG antibodies in the serum of maternal and umbilical cord blood throughout patient cohort.
