# Supplementary material for: Impact of SARS-CoV-2 Infection and Vaccination on Pregnancy Outcome and Passive Neonatal Immunity
Source: Cells. 2025 Nov 19;14(22):1812. doi: 10.3390/cells14221812 (PMC12651213; doi:10.3390/cells14221812)

Figure S1. NCP transfer ratio between maternal blood to umbilical cord blood in infected, acute infected and vaccinated/infected groups. Data is shown as mean ± SEM and analyzed using the Kruskal-Wallis test *p<0.05 **p<0.01

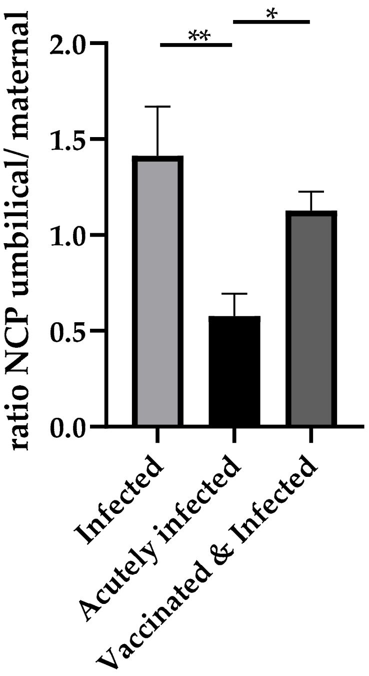

Supplement: Supplementary file 1 [file cells-14-01812-s001.zip › Figure S1.pdf]
