# Supplementary material for: Impact of SARS-CoV-2 Infection and Vaccination on Pregnancy Outcome and Passive Neonatal Immunity
Source: Cells. 2025 Nov 19;14(22):1812. doi: 10.3390/cells14221812 (PMC12651213; doi:10.3390/cells14221812)

Figure S2. Positivity for anti-CMV IgG in maternal blood samples across the study groups. Data are analyzed using the Kruskal-Wallis test and shown as mean ± SEM. *p<0.05.

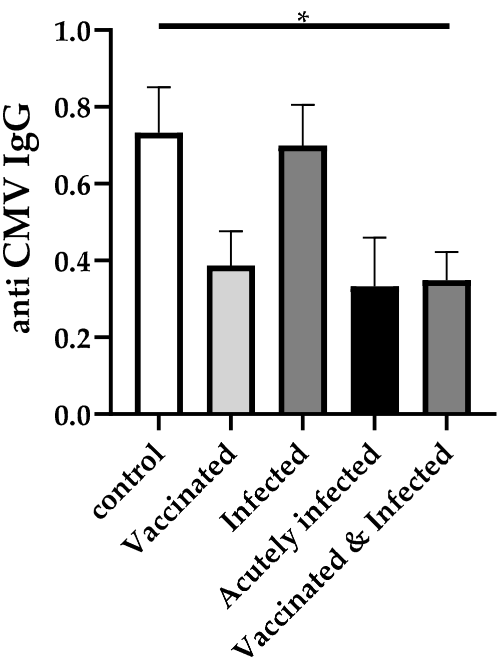

Supplement: Supplementary file 1 [file cells-14-01812-s001.zip › Figure S2.pdf]
