# Supplementary material for: Impact of SARS-CoV-2 Infection and Vaccination on Pregnancy Outcome and Passive Neonatal Immunity
Source: Cells. 2025 Nov 19;14(22):1812. doi: 10.3390/cells14221812 (PMC12651213; doi:10.3390/cells14221812)
Supplement: Supplementary file 1 [file cells-14-01812-s001.zip › Table S1.pdf]

Table S1. Number of pregnant women by time of infection.

	**Infected**	**Vaccinated & infected**
1st trimester (N)	4	9
2nd trimester (N)	9	18
3rd trimester (N)	8	21
