# Supplementary material for: Impact of SARS-CoV-2 Infection and Vaccination on Pregnancy Outcome and Passive Neonatal Immunity
Source: Cells. 2025 Nov 19;14(22):1812. doi: 10.3390/cells14221812 (PMC12651213; doi:10.3390/cells14221812)
Supplement: Supplementary file 1 [file cells-14-01812-s001.zip › Table S2.pdf]

Table S2. Number of pregnant women by time of vaccination.

	vaccinated			vaccinated and infected		
	**1st dose**	**2nd dose**	**3rd dose**	**1st dose**	**2nd dose**	**3rd dose**
1st trimester (N)	4	3	2	6	5	3
2nd trimester (N)	4	3	5	7	6	13
3rd trimester (N)	1	1	1			
