# Supplementary material for: Impact of SARS-CoV-2 Infection and Vaccination on Pregnancy Outcome and Passive Neonatal Immunity
Source: Cells. 2025 Nov 19;14(22):1812. doi: 10.3390/cells14221812 (PMC12651213; doi:10.3390/cells14221812)
Supplement: Supplementary file 1 [file cells-14-01812-s001.zip › Table S3.pdf]

Table S3. Levels of Anti-spike and presence of Anti-NCP antibodies in maternal and umbilical cord blood in correlation with the latest time point of maternal vaccination or infection. MB maternal blood, UB umbilical cord blood

Correlation p-value	MB Anti-spike IgG level	UB Anti-spike IgG level	MB Anti-NCP IgG+	UB Anti-NCP IgG+
**Latest vaccination (trimester)**	**<0.0001**	**<0.0001**	0.2872	0.8257
**Latest infection (trimester)**	0.0836	0.5211	**<0.0001**	**0.0024**
