# Supplementary material for: Impact of SARS-CoV-2 Infection and Vaccination on Pregnancy Outcome and Passive Neonatal Immunity
Source: Cells. 2025 Nov 19;14(22):1812. doi: 10.3390/cells14221812 (PMC12651213; doi:10.3390/cells14221812)
Supplement: Supplementary file 1 [file cells-14-01812-s001.zip › Table S4.pdf]

**Table S4.** Presence of anti-TORCH antibodies in maternal and umbilical cord blood. The number of positive patients out of the total number of patients and the corresponding percentages are given. MB, maternal blood, UB, umbilical cord blood.

| | control | vaccinated | infected | acute infected | vaccinated/ infected |
|---|---|---|---|---|---|
| **MB Toxo IgM positive (N; %)** | 3/15 20% | 6/32 18.8% | 1/21 4.8% | 0 | 6/45 13.3% |
| **UB Toxo IgM positive (N; %)** | 0 | 0 | 0 | 0 | 0 |
| **MB ROP1 IgM positive (N; %)** | 0 | 3/32 9.4% | 0 | 0 | 2/45 4.4% |
| **UB ROP1 IgM positive (N; %)** | 0 | 0 | 0 | 0 | 1/37 2.7% |
| **MB CMV IgM positive (N; %)** | 0 | 0 | 1/21 4.8% | 0 | 1/45 2.2% |
| **UB CMV IgM positive (N; %)** | 0 | 0 | 0 | 0 | 0 |
| **MB Toxo IgG positive (N; %)** | 4/15 37.5% | 12/33 36.4% | 3/21 14.3% | 4/15 26.7% | 15/45 33.3% |
| **UB Toxo IgG positive (N; %)** | 4/12 33.3% | 6/23 26.14% | 1/16 6.3% | 4/11 36.4% | 11/37 29.7% |
| **MB Rubella IgG positive (N; %)** | 13/15 86.7% | 31/33 93.9% | 19/21 90.5% | 13/13 100% | 43/45 95.6% |
| **UB Rubella IgG positive (N; %)** | 12/12 100% | 23/23 100% | 15/16 93.8% | 11/11 100% | 37/37 100% |
| **MB CMV IgG positive (N; %)** | 11/15 73.3% | 12/33 38.7% | 14/21 66.7% | 5/15 33.3% | 15/45 33.3% |
| **UB CMV IgG (N; %)** | 7/12 58.3% | 7/23 30.4% | 9/16 56.3% | 3/12 25% | 14/37 37.8% |
| **MB HSV1 IgG positive (N; %)** | 11/15 75% | 20/32 62.5% | 16/21 76.2% | 12/15 80% | 30/45 66.7% |
| **UB HSV1 IgG positive (N; %)** | 9/12 75% | 15/23 65.2% | 12/16 75% | 8/11 72.7% | 24/37 64.9% |
| **MB HSV2 IgG positive (N; %)** | 3/15 20% | 1/33 3% | 2/21 9.5% | 1/15 6.7% | 3/45 6.7% |
| **UB HSV2 IgG positive (N; %)** | 2/12 16.7% | 1/23 4.3% | 1/16 6.3% | 0/11 0% | 2/37 5.4% |
