# Supplementary material for: Impact of SARS-CoV-2 Infection and Vaccination on Pregnancy Outcome and Passive Neonatal Immunity
Source: Cells. 2025 Nov 19;14(22):1812. doi: 10.3390/cells14221812 (PMC12651213; doi:10.3390/cells14221812)
Supplement: Supplementary file 1 [file cells-14-01812-s001.zip › Table S5.pdf]

Table S5. Correlation of appearance of IgG TORCH antibodies in the serum of maternal and umbilical cord blood throughout patient´ cohort. (M=maternal serum, UB= umbilical cord blood serum, Toxo= *Toxoplasma-gondii*, Rub= Rubella, CMV= Cytomegalovirus, HSV= Herpes simplex virus). Data shown as p-value (Spearman r).

	**UB IgG**
MB Toxo IgG	**<0.0001 (1.000)**
MB Rub IgG	**(1.000)**
MB CMV IgG	**<0.0001 (0.9658)**
MB HSV1 IgG	**<0.0001 (0.9123)**
MB HSV2 IgG	**<0.0001 (1.000)**
